# Effects of ultrasound pregnancy dating on neonatal morbidity in late preterm and early term male infants: a register-based cohort study

**DOI:** 10.1186/s12884-016-1129-z

**Published:** 2016-10-31

**Authors:** Merit Kullinger, Bengt Haglund, Helle Kieler, Alkistis Skalkidou

**Affiliations:** 1 Department of Women’s and Children’s Health, Uppsala University, Uppsala, Sweden; 2 Centre for Clinical Research, Västmanland County Hospital, Västerås, Sweden; 3 Department of Medicine, Solna, Centre for Pharmacoepidemiology (CPE), Karolinska Institute, Stockholm, Sweden; 4 Department of Obstetrics and Gynecology, Västmanland County Hospital, Västerås, Sweden

**Keywords:** Pregnancy dating, Ultrasound, Gestational age, Antenatal, Infant, Morbidity

## Abstract

**Background:**

Assessing gestational age by ultrasound can introduce a systematic bias due to sex differences in early growth.

**Methods:**

This cohort study included data on 1,314,602 births recorded in the Swedish Medical Birth Register. We compared rates of prematurity-related adverse outcomes in male infants born early term (gestational week 37–38) or late preterm (gestational week 35–36), in relation to female infants, between a time period when pregnancy dating was based on the last menstrual period (1973–1978), and a time period when ultrasound was used for pregnancy dating (1995–2010), in order to assess the method’s influence on outcome by fetal sex.

**Results:**

As expected, adverse outcomes were lower in the later time period, but the reduction in prematurity-related morbidity was less marked for male than for female infants. After changing the pregnancy dating method, male infants born early term had, in relation to female infants, higher odds for pneumothorax (Cohort ratio [CR] 2.05; 95 % confidence interval [CI] 1.33–3.16), respiratory distress syndrome of the newborn (CR 1.99; 95 % CI 1.33–2.98), low Apgar score (CR 1.26; 5 % CI 1.08–1.47), and hyperbilirubinemia (CR 1.12; 95 % CI 1.06–1.19), when outcome was compared between the two time periods. A similar trend was seen for late preterm male infants.

**Conclusion:**

Misclassification of gestational age by ultrasound, due to size differences, can partially explain currently reported sex differences in early term and late preterm infants’ adverse neonatal outcomes, and should be taken into account in clinical decisions and when interpreting study results related to fetal sex.

**Electronic supplementary material:**

The online version of this article (doi:10.1186/s12884-016-1129-z) contains supplementary material, which is available to authorized users.

## Background

The use of ultrasound (US) has an unquestionable role in modern obstetrical practice. In many countries, as in Sweden, fetal biometry is the recommended single method for estimation of gestational age (GA) and estimated delivery date (EDD), and the date of the last menstrual period (LMP) is only used when no US estimate is available [1]. In other countries, the estimation is based on the date of the LMP or a combination of both methods.

Biometry using US in the first or second trimester is generally a more precise method for assessing GA and pregnancy dating, than estimates based on the date of the LMP [2]. Estimates are more precise when they are based on first-trimester rather than second-trimester biometry [3, 4]. Early differences in fetal growth do exist [5–7], but it has been assumed that individual variation is too small to have any clinical significance [8]. Recent studies challenge this assumption [9–12]. For example, second-trimester US dating underestimates GA and overestimates preterm delivery rates in infants born small for gestational age (SGA) [12]. Furthermore, postponing the date of estimated delivery by 7 days by US is associated with birth of SGA infants [11], increased risk of stillbirth, low Apgar score, and neonatal death [10].

The reported sex differences in early fetal growth [7, 8] can be used as one example among many other variables possibly affecting the accuracy of US-based estimation of GA. In a study by Skalkidou et al., increased mortality and morbidity in post-term female infants in relation to male infants was seen after US was introduced in Sweden as the method for assessing GA [9]. This increase in mortality and morbidity can reflect the fact that girls born post-term may be more mature than their US-based GA indicates, as their EDD was moved forward in time because of their smaller size at the time of ultrasound pregnancy dating.

According to this hypothesis, male fetuses could be less mature than the US-based GA estimate, since the approximation of GA from fetal size used during ultrasound dating would not consider size differences. In Sweden, a second trimester scan, using the biparietal diameter (BPD)-measurement for pregnancy dating, is typically performed around gestational week 18 at what time the mean difference (male vs. female) in BPD is considered to be 1 mm [5]. An introduced bias in the GA estimate, due to size difference by fetal sex at the time of pregnancy dating, would be hypothesized to affect clinical management and neonatal outcomes in the late preterm and early term period. Although often treated as term, late preterm infants more commonly present with prematurity-related morbidity such as hyperbilirubinemia, respiratory distress syndrome (RDS) of the newborn, transient tachypnea of the newborn, interventions to support breathing, and readmissions for hospital care [13].

Our hypothesis was that a change in the method of dating pregnancy might have led to an increased risk for prematurity-related adverse outcomes among male infants in relation to female infants by introducing a misclassification bias due to sex differences in early growth. The aim of this study was to compare rates of adverse prematurity-related outcomes in early term and late preterm male infants in relation to their female counterparts, between a time period, when pregnancy dating was based on the LMP, and a time period when ultrasound was used for pregnancy dating, in order to assess the dating method’s influence on prematurity-related adverse outcomes by fetal sex.

## Methods

In this study, we used data on 1,314,602 births in Sweden to compare adverse outcomes related to prematurity between male and female infants by method of pregnancy dating (US or from LMP). We compared prevalence of outcomes between sexes in a time period when LMP was used as the only method for dating pregnancies (1973–1978) and similarly the prevalence of outcomes were compared between sexes after US was introduced as the method for dating pregnancies (1995–2010). The risk estimates, by fetal sex, that were generated for each of the two time periods were then compared.

Information was collected from the Swedish Medical Birth Register (MBR), which contains information on more than 99 % of all births in Sweden since 1973. For the purposes of the current study, the period between 1978 and 1995 was not included since policies on the method for pregnancy dating and registration of the US-based EDD were not uniform throughout the country. The register includes data about maternal socio-demographic characteristics and prospectively collected information during pregnancy, delivery, and the neonatal period (first 28 days) [14]. The register has been evaluated as reliable for research purposes, with good internal validity [15]. Diagnoses are classified and recorded by the treating physician or midwife according to the International Classification of Diseases (ICD). The version used during 1973–1978 was ICD-8, while during 1995–2010 the versions were ICD-9 and ICD-10.

Up to 1978, practically all clinics were using the LMP date in order to calculate the EDD. With the introduction of US in Sweden, the practice was changed to estimate the EDD from second-trimester US biometry irrespective of the LMP date [1]. Before 1980, fewer than 5 % of hospitals practiced US scanning. By contrast, from 1995 on, nearly all clinics based EDD assessment on US biometry and documented the EDD-US in the MBR. Routine US scanning has been offered to all pregnant women since 1990, and more than 95 % of the women accept this offer [14]. Such routine scanning is typically performed early in the second trimester (gestational weeks 17–19) for assessing GA, detection of multiple births, placental location, and congenital anomalies [1]. According to a 1996 study, when 59 clinics in Sweden provided obstetric and antenatal care, US scanning was performed at gestational weeks 16–20 in 52 clinics and at 10–15 weeks in three clinics [1]. Since then, the use of first-trimester US has increased gradually.

Our study population consisted of two cohorts including all singleton births in Sweden, with valid birth dates for both mother and infant, from 1973 to 1978 (GA assessment based on the LMP date) and from 1995 to 2010 (GA assessment by US).

Information was retrieved on GA at delivery (based on LMP date and US assessment, respectively), the level of the hospital, maternal age, parity, infant sex, Apgar score [16], any neonatal deaths, and diagnoses of adverse outcomes related to late prematurity. Diagnoses that allowed comparison between the ICD versions and with higher incidence among premature infants were chosen. These were: pneumothorax P25 (ICD-10), 7702 (ICD-9), 77620, and 77625 (ICD-8); RDS P22.0 (ICD-10), 769 (ICD-9), and 7761 (ICD-8); other respiratory conditions such as P22.1, P22.8, P22.9, P28.3, P28.4 (ICD-10), 7706, 7708 (ICD-9), 77629, 77699, and 77680 (ICD-8); hyperbilirubinemia P55, P57, P58, P59 (ICD-10), 7730, 7731, 7732, 7734, 7735, 774 (ICD-9), 774, 775, and 77893 (ICD-8) [17–21].

For the purposes of this study, infants were classified into three groups: those born from 39 weeks + 0 days to 40 weeks + 6 days (273–286 days, designated midterm); from 37 weeks + 0 days to 38 weeks + 6 days (259–272 days, designated early term); and from 35 weeks + 0 days to 36 weeks + 6 days (245–258 days, hereby referred to as late preterm). We chose to use 2-week intervals to create groups of equal length for comparison. The common definition of late preterm infants also includes infants born from 34 weeks + 0 days to 34 weeks + 6 days (238–244 days), but these infants were not included in the current study in order to reduce possible misclassification by the inclusion of moderately preterm infants.

Data on the study population were cross-tabulated by infant sex, GA, and the two time periods (1973–1978 and 1995–2010). As a first step, rates of adverse outcomes per 1000 live births were calculated according to infant sex, GA, and the studied period. Thereafter, for each time period, using the gestational age categories as the exposure variable (early term and late preterm infants, respectively, with midterm infants as the reference category), we used logistic regression to estimate odds for prematurity-related adverse outcomes such as neonatal death, low Apgar score (<7 at 5 min), pneumothorax, RDS, other respiratory conditions, and hyperbilirubinemia. Then, for each time period, odds ratios (ORs) with 95 % confidence intervals (CIs) for adverse prematurity-related outcomes among male infants, always in relation to their female counterparts, were calculated with logistic regression, separately for early term and for late preterm infants. Finally, to estimate the change in male risk before and after the introduction of ultrasound pregnancy dating, the relative change in odds for adverse prematurity-related outcomes between the two periods, among male infants in the early term and late preterm groups in relation to their female counterparts, was calculated as the ratio between the ORs for each period (Cohort Ratio, CR). The multivariate analyses were adjusted for maternal age, parity, and level of hospital care.

The statistical analyses were conducted using the SAS 9.3 software package (SAS Institute, Cary, NC, USA). The ORs and CRs with their 95 % confidence intervals were estimated with the GENMOD procedure.

## Results

The characteristics of the study cohorts are presented in Table 1, with the number of male and female infants born in each GA group during 1973–1978 and 1995–2010. The proportion of midterm births (74.6 % in the early vs. 70.5 % in the late period) was higher in the early cohort, and the proportion of early term births (21.0 % in the early vs. 25.5 % in the late period) was lower in the early cohort. The proportion of late preterm births was similar in the two cohorts (4.3 % in the early vs. 4.0 % in the late period). Mean maternal age for the first time period (1973–1978) was 27 years (SD = 5), and mean maternal age in the later time period (1995–2010) was 30 (SD = 5).

**Table 1:** Number and distribution of two cohorts of singleton births in Sweden, with mean and standard deviation (SD) of birthweight, birth length, and Apgar score by infant sex and gestational age, before and after the implementation of ultrasound for pregnancy dating

		1973–1978				1995–2010			
		Male		Female		Male		Female	
	Gestational age	N	%	N	%	N	%	N	%
	35–40 weeks	187,163	100.0	170,375	100.0	479,118	100.0	477,946	100.0
	35–36 weeks	8,737	4.7	6,733	4.0	20,527	4.3	18,095	3.8
	37–38 weeks	41,434	22.1	33,746	19.8	122,074	25.5	121,500	25.4
	39–40 weeks	136,992	73.2	129,896	76.2	336,517	70.2	338,351	70.8
Gestational age	Infant characteristics	Mean	SD	Mean	SD	Mean	SD	Mean	SD
35–36 weeks	Birthweight (g)	2,850	499	2,749	503	2,846	452	2,778	461
	Birth length (cm)	47.9	2.5	47.3	2.4	47.6	2.2	47.0	2.2
	Apgar score	9.2	1.3	9.2	1.3	9.5	1.0	9.6	1.0
37–38 weeks	Birthweight (g)	3,284	472	3,159	465	3,349	460	3,254	450
	Birth length (cm)	49.7	2.1	48.9	2.1	49.6	2.0	48.9	2.0
	Apgar score	9.5	0.9	9.5	0.9	9.8	0.7	9.8	0.6
39–40 weeks	Birthweight (g)	3,591	463	3,451	446	3,665	451	3,544	437
	Birth length (cm)	51.0	2.0	50.1	2.0	51.0	1.9	50.2	1.8
	Apgar score	9.6	0.8	9.6	0.7	9.8	0.7	9.8	0.6

Table 2 demonstrates rates of prematurity-related adverse outcomes by sex and gestational age in the two time periods. The ORs for adverse outcomes are presented with midterm infants of the same sex as the reference category. Rates of adverse neonatal outcomes were generally lower for both male and female infants in the later period. Rates of adverse outcomes were lower for female infants than for male infants, in both time periods, with the exception of neonatal death among late preterm infants in the later period. ORs for prematurity-related outcomes were lower for early term infants, than for late preterm infants, as expected.

**Table 2:** Rates and adjusted^a^ odds ratios (OR) with 95 % confidence intervals (95 % CI) for prematurity-related adverse outcomes by gestational age (GA) at birth among infants born at 35–36 weeks or 37–38 weeks, compared with 39–40 weeks, before and after the implementation of ultrasound in Sweden for pregnancy dating

Outcome by GA at Birth (weeks)	1973–1978				1995–2010			
	Male infants		Female infants		Male infants		Female infants	
	Rate/1000	OR (95 % CI)	Rate/1000	OR (95 % CI)	Rate/1000	OR (95 % CI)	Rate/1000	OR (95 % CI)
Neonatal death								
35–36	20.7	10.9 (8.97–13.1)	18.1	11.0 (8.81–13.8)	4.9	9.45 (7.39–12.1)	5.2	11.0 (8.48–14.2)
37–38	5.6	2.88 (2.42–3.44)	4.4	2.62 (2.13–3.24)	1.3	2.49 (2.01–3.09)	1.0	2.01 (1.58–2.55)
39–40	1.9	1.00 (ref)	1.7	1.00 (ref)	0.5	1.00 (ref)	0.5	1.00 (ref)
Apgar score <7 at 5 min								
35–36	40.7	4.61 (4.08–5.21)	37.9	5.25 (4.55–6.06)	24.8	2.99 (2.71–3.29)	19.8	3.02 (2.69–3.38)
37–38	14.4	1.63 (1.48–1.80)	13.5	1.88 (1.68–2.11)	9.0	1.13 (1.06–1.22)	6.7	1.08 (0.99–1.17)
39–40	8.8	1.00 (ref)	7.2	1.00 (ref)	7.9	1.00 (ref)	6.2	1.00 (ref)
Pneumothorax								
35–36	7.6	8.87 (6.53–12.0)	4.5	7.82 (5.10–12.0)	6.7	3.92 (3.25–4.73)	3.0	2.99 (2.24–3.99)
37–38	2.0	2.32 (1.74–3.08)	1.4	2.41 (1.66–3.50)	2.3	1.41 (1.22–1.63)	0.8	0.83 (0.66–1.04)
39–40	0.8	1.00 (ref)	0.5	1.00 (ref)	1.6	1.00 (ref)	0.9	1.00 (ref)
RDS^b^								
35–36	30.6	33.1 (26.8–41.0)	17.8	35.6 (26.3–48.2)	16.2	82.6 (63.6–107)	8.6	63.6 (45.9–88.3)
37–38	3.7	3.91 (3.09–4.95)	2.6	5.09 (3.69–7.01)	1.3	6.33 (4.76–8.42)	0.5	3.22 (2.18–4.75)
39–40	0.9	1.00 (ref)	0.5	1.00 (ref)	0.2	1.00 (ref)	0.1	1.00 (ref)
Other respiratory conditions								
35–36	49.2	6.44 (5.74–7.22)	38.0	7.18 (6.20–8.31)	100.0	7.07 (6.70–7.46)	65.3	6.50 (6.08–6.96)
37–38	18.9	2.42 (2.21–2.66)	12.0	2.22 (1.96–2.51)	27.6	1.87 (1.79–1.95)	15.4	1.51 (1.43–1.60)
39–40	7.8	1.00 (ref)	5.4	1.00 (ref)	14.9	1.00 (ref)	10.2	1.00 (ref)
Hyperbilirubinemia								
35–36	238.5	5.88 (5.56–6.21)	219.5	7.62 (7.14–8.13)	292.7	16.5 (15.9–17.1)	261.2	18.8 (18.0–19.6)
37–38	113.4	2.41 (2.32–2.51)	98.5	2.97 (2.84–3.11)	53.0	2.33 (2.25–2.41)	40.9	2.39 (2.30–2.48)
39–40	50.0	1.00 (ref)	35.2	1.00 (ref)	23.5	1.00 (ref)	17.6	1.00 (ref)

^a^ Adjusted for maternal age, parity, and level of hospital care

^b^ Respiratory distress syndrome of the newborn

Table 3 includes the ORs for prematurity-related adverse outcomes among early term male infants, with female infants as reference category, in the two time periods. ORs for adverse outcomes were higher for all comparisons in relation to female infants. Comparing the two time periods, ORs for male infants were increased in the second study period, as reflected by the statistically significant cohort ratios (CR). Between the two time periods, there was an increase in odds for early term male infants, in relation to females, for pneumothorax (CR 2.05, CI 1.33–3.16), RDS (CR 1.99, CI 1.33–2.98), a low Apgar score (CR 1.26, CI 1.08–1.47), other respiratory conditions (CR 1.14, CI 1.00–1.30), and hyperbilirubinemia (CR 1.12, CI 1.06–1.19) in the later time period. There was no significant change in cohort ratios for perinatal mortality (Table 3).

**Table 3:** Adjusted^a^ odds ratios (OR) with 95 % confidence intervals (95 % CI) for prematurity-related adverse outcomes by male sex at birth in gestational week 37–38, and cohort ratios (CR) for the change in male risk before and after the implementation of ultrasound for pregnancy dating in Sweden

Outcome	Birth Cohort 1973–1978 Odds Ratio^ab^ (95 % CI)	Birth Cohort 1995–2010 Odds Ratio^ab^ (95 % CI)	Cohort Ratios: Ratio of Male ORs 1995–2010 to 1973–1978 (95 % CI)
Neonatal death	1.28 (1.04–1.58)	1.36 (1.07–1.73)	1.06 (0.77–1.45)
Apgar score <7 at 5 min	1.07 (0.94–1.21)	1.35 (1.23–1.48)	1.26 (1.08–1.47)
Pneumothorax	1.44 (1.00–2.06)	2.95 (2.34–3.72)	2.05 (1.33–3.16)
RDS^c^	1.44 (1.11–1.87)	2.86 (2.11–3.89)	1.99 (1.33–2.98)
Other respiratory conditions	1.59 (1.41–1.80)	1.81 (1.71–1.92)	1.14 (1.00–1.30)
Hyperbilirubinemia	1.17 (1.12–1.23)	1.31 (1.26–1.36)	1.12 (1.06–1.19)

^a^ Adjusted for maternal age, parity, and level of hospital care

^b^ The reference category was female infants

^c^ Respiratory distress syndrome of the newborn

In Table 4, the corresponding ORs, but instead in late preterm male infants, are presented. The ORs for adverse outcomes for male late preterm infants in relation to females were significantly higher for all outcomes in both time periods, except for neonatal mortality. Late preterm male infants in comparison to females had increased risks for other respiratory conditions in the latter time period (CR 1.22, CI 1.02–1.45). Despite not reaching statistical significance, all other CRs for neonatal morbidity were positive for late preterm infants, following the same trend as in early term infants.

**Table 4:** Adjusted^a^ odds ratios (OR) with 95 % confidence intervals (95 % CI) for prematurity-related adverse outcomes by male sex at birth in gestational week 35–36, and cohort ratios (CR) for the change in male risk before and after the implementation of ultrasound for pregnancy dating in Sweden

Outcome	Birth Cohort 1973–1978 Odds Ratio^a,b^ (95 % CI)	Birth Cohort 1995–2010 Odds Ratio^a,b^ (95 % CI)	Cohort Ratios: Ratio of Male ORs 1995–2010 to 1973–1978 (95 % CI)
Neonatal death	1.15 (0.91–1.45)	0.95 (0.71–1.26)	0.82 (0.57–1.19)
Apgar score <7 at 5 min	1.08 (0.91–1.28)	1.26 (1.10–1.45)	1.17 (0.94–1.45)
Pneumothorax	1.70 (1.10–2.62)	2.27 (1.66–3.11)	1.34 (0.78–2.28)
RDS^c^	1.74 (1.40–2.16)	1.89 (1.56–2.28)	1.08 (0.81–1.45)
Other respiratory conditions	1.31 (1.12–1.54)	1.60 (1.48–1.72)	1.22 (1.02–1.45)
Hyperbilirubinemia	1.12 (1.03–1.20)	1.18 (1.12–1.23)	1.05 (0.96–1.15)

^a^ Adjusted for maternal age, parity, and level of hospital care

^b^ The reference category was female infants

^c^ Respiratory distress syndrome of the newborn

There were practically no differences in the results when births by cesarean section delivery were excluded (Additional file 1).

## Discussion

After the introduction of US for pregnancy dating in Sweden, the risk of prematurity-related adverse outcomes was increased among male infants born at early term relative to that of female infants. A similar trend was observed for late preterm male infants. The relative increase in male risks was in line with a less marked reduction of prematurity-related morbidity among male than among female infants in the later time period. This was also consistent with the hypothesized introduction of a misclassification bias after the introduction of US-biometry as the method of assessing GA. As male fetuses are usually larger than their female counterparts, they may be considered older at the time of US dating and their EDD will be moved backward in time. Thus, at birth, male infants would be less mature and at greater risk for prematurity-related adverse outcomes.

The assessment of GA by US has had beneficial effects, such as a reduction in the need for the induction of labor for prolonged pregnancy [22] and improved pregnancy outcomes [23]. However, the adverse effects of US pregnancy dating also need to be considered. Variations in early growth related to fetal or maternal factors leading to misclassification of GA can affect morbidity and mortality in selected groups, such as post-term female infants [9]. In a large study comparing the GA based on second-trimester US biometry with the GA based on the date of embryo transfer after in vitro fertilization (IVF), an underestimation of GA with US biometry was observed among infants later diagnosed as SGA [6]. These results were replicated in the Extremely Preterm Infants in Sweden Study, in which a discrepancy of seven days or more between US-based and LMP-based estimation of GA was associated with an increased risk of being born SGA [24]. Similarly, fetal sex, which affects the assessment of GA by US [5, 12], could also affect perinatal outcomes, consistent with the results of this study.

We found that there were more significant increases in odds for adverse outcomes among early term male infants than among late preterm ones. An explanation for this finding could be that routines for neonatal care depend on the assessed GA. Concerns have been expressed regarding the lack of awareness of the special needs for care of late preterm infants compared with the intense management efforts for more preterm ones [13]. This reasoning could also be applied when comparing early term and late preterm infants. Early term infants, that were actually late preterm, would be expected to cope well and would receive less attention than needed, whereas late preterm infants, that were actually more preterm, would anyway receive active care from birth. There were more children born early term than late preterm, which reduced to some extent the power for analyses of outcome in the late preterm group.

By focusing on comparisons of male relative to female risks in both study periods, we tried to minimize the risk of bias. With this design, a possible bias would have had to selectively affect the outcomes for either male or female infants, and be more prevalent in only one of the study periods, to affect the outcome of the final analyses. The improvements in neonatal medicine during the end of the last century have actually favored boys to a higher degree than girls, possibly because of their excess inherent vulnerability and higher risk for prematurity [25]. During the two time periods in this study, maternal age at childbirth increased, which should not have affected the outcome variables in a sex-specific way. Further, maternal obesity rates increased in the general population during the study period, but there is no known association of obesity with infant sex. The Swedish MBR did not include the variable ‘weight at first antenatal visit’ until 1992, and therefore information on weight or body mass index could not be adjusted for in the analyses, nor was information on smoking available for comparison. Smoking decreased during the study periods, but it is not thought to affect prematurity-related outcomes in a sex-specific way. An increasing proportion of cesarean deliveries is affecting male infants to a higher degree in some studies [26, 27]. Cesarean section could not act as a true confounder, as is does not affect infant sex (exposure) but only the outcome. When cesarean deliveries were excluded from analyses, there were only minimal differences in the results. The lower neonatal mortality in the more recent cohort, as well as the narrowing of the mortality gap between male and female infants, are consistent with other reports [28, 29].

Changes in the classification of diagnoses in the three different versions of the ICD system limited the numbers of diagnoses that could be included in the analyses. Other possible limitations to the study were changes in diagnosis registration and clinical management guidelines between the two periods, which could apply to pneumothorax, hyperbilirubinemia, and respiratory conditions. Such changes could have affected male and female infants differently, although medical advances would more likely have favored male infants, and would have only attenuated the associations found in this study. It is unlikely that diagnoses were recorded differently by sex of the infant, and, moreover, it could only have introduced a bias in the registered outcomes if this occurred in one, and not the other, of the studied time periods. One of the major strengths of this work was that the current study’s results are in line with our and others previous studies’ hypothesis on possible misclassification by fetal sex, as increased odds for prematurity-related outcomes among late preterm and early term male infants is consistent with increased risks for postmaturity-related adverse outcome in post-term female infants [7, 9]. Another major strength of this register-based study was the large study population, with adequate power for the statistical analyses. The MBR includes practically all births in Sweden since 1973, which accounts for high external validity. However, only singleton pregnancies are included in this study. When considering wider external validity, it can be argued that population-based differences in fetal growth exist, which can be attributed both to genetic, medical, and social factors. However, in the Intergrowth-project, the fetal skeletal measurements were similar among the included healthy and well-nourished women from eight geographically diverse populations [30]. During the later period, US dating in the first trimester instead of the second trimester increased in Sweden. Nevertheless, this could only have led to an attenuation of the observed associations, as measurements in earlier gestation have less variance and would have reduced the degree of misclassification of GA [3, 4].

Gestational length at delivery strongly correlates with neonatal outcome, and the estimated gestational length is important for clinical decision-making. Therefore, it is important to evaluate the methods for assessing GA critically, and to identify fetuses that deviate sufficiently from the mean to affect neonatal outcome. The sex differences in fetal size in the second trimester are usually small, corresponding to a few days difference in the EDD [8]. The significant effects on prematurity-related outcomes in our study are thus likely to be attributed to those few fetuses with large discrepancies between the actual and US-estimated GA.

In the clinical setting, when US biometry is used for pregnancy dating, one should take into consideration biological [6] and methodological [31] variance. When there is a large discrepancy between LMP date- and US-based estimations [10], clinicians should try to combine available data in order to get the best estimate of GA before clinical decision-making. For example, for pregnancies after IVF, the day of embryo transfer is often preferred to US biometry for assessing GA. In other cases, pregnant women can sometimes provide precise information on the days around which conception took place, which can be of added value. As the variance in growth increases with GA, a first-trimester estimation of GA would be preferred to a second-trimester scan for the purpose of pregnancy dating [3, 4]. The misclassification bias due to sex differences in fetal growth [9] can be reduced using first-trimester US biometry [32], and an increasing use of first trimester ultrasound in Sweden will probably improve precision of GA estimates which would help to optimize neonatal care. However, one should bear in mind that even such assessments can be biased by early growth restriction [33]. A large discrepancy between the estimates derived from US-examination and the date of LMP might constitute an indication for a repeat scan, as it suggests higher odds for adverse outcomes such as early growth restriction [34].

## Conclusions

There was a significant reduction of infant morbidity between the two study periods, as expected. However, male infants on the edge of prematurity have not benefitted as much as female infants in terms of prematurity-related outcomes. This might reflect a bias introduced by the pregnancy dating method, as a proportion of male infants might be more premature at birth than estimated and thus more prone to prematurity-related adverse outcomes. Misclassification of GA because of sex differences in fetal size could thus partially explain the currently reported male disadvantage in neonatal outcomes of early term and late preterm infants, and should be taken into account in clinical decisions and when interpreting study results related to fetal sex.

## Additional file

Additional file 1: Results from the stratified analysis by mode of delivery. (DOCX 101 kb)

## Abbreviations

BPD: Biparietal diameter
CI: Confidence interval
CR: Cohort ratio
EDD: Estimated delivery date
GA: Gestational age
ICD: International classification of diseases
IVF: In vitro fertilization
LMP: Last menstrual period
MBR: Swedish Medical Birth Register
OR: Odds ratio
RDS: Respiratory distress syndrome of the newborn
SGA: Small for gestational age
US: Ultrasound
