# Supplementary material for: Effects of ultrasound pregnancy dating on neonatal morbidity in late preterm and early term male infants: a register-based cohort study
Source: BMC Pregnancy Childbirth. 2016 Oct 31;16:335. doi: 10.1186/s12884-016-1129-z (PMC5088647; doi:10.1186/s12884-016-1129-z)
Supplement: Additional file 1: — Results from the stratified analysis by mode of delivery. (DOCX 101 kb) [file 12884_2016_1129_MOESM1_ESM.docx]

**Additional file –** Results from the stratified analysis by mode of delivery

**Solely vaginal deliveries –** Adjusted^a^ odds ratios (OR) with 95% confidence intervals (95% CI) for prematurity-related adverse outcomes by male sex at birth in **gestational week 37–38**, and cohort ratios (CR) for the change in male risk before and after the implementation of ultrasound for pregnancy dating in Sweden

| Outcome | Birth Cohort  1973–1978  Odds Ratio^a,b^  (95% CI) | Birth Cohort  1995–2010  Odds Ratio^a,b^  (95% CI) | Cohort Ratios:  Ratio of Male ORs,  1995–2010 to 1973–1978  (95% CI) |
| --- | --- | --- | --- |
| Neonatal death | 1.22 (0.95 - 1.56) | 1.30 (0.95 - 1.78) | 1.07 (0.71 - 1.60) |
| Apgar score <7 at 5 min | 1.16 (0.99 - 1.35) | 1.37 (1.21 - 1.55) | 1.19 (0.98 - 1.45) |
| Pneumothorax | 1.13 (0.72 - 1.78) | 3.01 (2.06 - 4.39) | 2.66 (1.48 - 4.80) |
| RDS^c^ | 1.42 (0.96 - 2.08) | 3.07 (1.85 - 5.09) | 2.17 (1.15 - 4.09) |
| Other respiratory conditions | 1.73 (1.50 - 2.01) | 1.70 (1.56 - 1.86) | 0.98 (0.83 - 1.16) |
| Hyperbilirubinemia | 1.18 (1.12 - 1.24) | 1.35 (1.30 - 1.41) | 1.15 (1.08 - 1.23) |

^a^ Adjusted for maternal age, parity, and level of hospital care

^b^ The reference category was female infants

^c^ Respiratory distress syndrome of the newborn

**Solely vaginal deliveries** – Adjusted^a^ odds ratios (OR) with 95% confidence intervals (95% CI) for prematurity-related adverse outcomes by male sex at birth in **gestational week 35–36**, and cohort ratios (CR) for the change in male risk before and after the implementation of ultrasound for pregnancy dating in Sweden

| Outcome | Birth Cohort  1973–1978  Odds Ratio^a,b^  (95% CI) | Birth Cohort  1995–2010  Odds Ratio^a,b^  (95% CI) | Cohort Ratios:  Ratio of Male ORs,  1995–2010 to 1973–1978  (95% CI) |
| --- | --- | --- | --- |
| Neonatal death | 1.15 (0.86 - 1.53) | 0.73 (0.48 - 1.11) | 0.64 (0.38 - 1.06) |
| Apgar score <7 at 5 min | 1.15 (0.93 - 1.42) | 1.23 (1.00 - 1.51) | 1.06 (0.79 - 1.43) |
| Pneumothorax | 2.30 (1.25 - 4.21) | 1.97 (1.31 - 2.96) | 0.86 (0.41 - 1.78) |
| RDS^c^ | 2.11 (1.56 - 2.86) | 2.13 (1.59 - 2.86) | 1.01 (0.66 - 1.54) |
| Other respiratory conditions | 1.36 (1.13 - 1.64) | 1.80 (1.62 - 2.00) | 1.32 (1.06 - 1.64) |
| Hyperbilirubinemia | 1.14 (1.05 - 1.24) | 1.15 (1.09 - 1.21) | 1.01 (0.92 - 1.11) |

^a^ Adjusted for maternal age, parity, and level of hospital care

^b^ The reference category was female infants

^c^ Respiratory distress syndrome of the newborn

**Solely cesarean section deliveries –** Adjusted^a^ odds ratios (OR) with 95% confidence intervals (95% CI) for prematurity-related adverse outcomes by male sex at birth in **gestational week 37–38**, and cohort ratios (CR) for the change in male risk before and after the implementation of ultrasound for pregnancy dating in Sweden

| Outcome | Birth Cohort  1973–1978  Odds Ratio^ab^  (95% CI) | Birth Cohort  1995–2010  Odds Ratio^ab^  (95% CI) | Cohort Ratios:  Ratio of Male ORs,  1995–2010 to 1973–1978  (95% CI) |
| --- | --- | --- | --- |
| Neonatal death | 1.53 (1.05 - 2.21) | 1.43 (1.00 - 2.06) | 0.94 (0.56 - 1.58) |
| Apgar score <7 at 5 min | 0.96 (0.78 - 1.19) | 1.30 (1.14 - 1.49) | 1.35 (1.05 - 1.74) |
| Pneumothorax | 2.27 (1.23 - 4.22) | 2.86 (2.13 - 3.84) | 1.26 (0.63 - 2.50) |
| RDS^c^ | 1.57 (1.09 - 2.25) | 2.69 (1.82 - 3.95) | 1.72 (1.01 - 2.92) |
| Other respiratory conditions | 1.39 (1.12 - 1.72) | 1.88 (1.74 - 2.03) | 1.35 (1.08 - 1.70) |
| Hyperbilirubinemia | 1.06 (0.91 - 1.23) | 1.17 (1.07 - 1.28) | 1.11 (0.93 - 1.32) |

^a^ Adjusted for maternal age, parity, and level of hospital care

^b^ The reference category was female infants

^c^ Respiratory distress syndrome of the newborn

**Solely cesarean section deliveries –** Adjusted^a^ odds ratios (OR) with 95% confidence intervals (95% CI) for prematurity-related adverse outcomes by male sex at birth in **gestational week 35–36**, and cohort ratios (CR) for the change in male risk before and after the implementation of ultrasound for pregnancy dating in Sweden

| Outcome | Birth Cohort  1973–1978  Odds Ratio^a,b^  (95% CI) | Birth Cohort  1995–2010  Odds Ratio^a,b^  (95% CI) | Cohort Ratios:  Ratio of Male ORs,  1995–2010 to 1973–1978  (95% CI) |
| --- | --- | --- | --- |
| Neonatal death | 1.17 (0.80 - 1.73) | 1.22 (0.83 - 1.79) | 1.04 (0.60 - 1.79) |
| Apgar score <7 at 5 min | 0.99 (0.75 - 1.31) | 1.35 (1.12 - 1.63) | 1.37 (0.98 - 1.91) |
| Pneumothorax | 1.19 (0.63 - 2.26) | 2.83 (1.72 - 4.64) | 2.37 (1.06 - 5.31) |
| RDS^c^ | 1.43 (1.04 - 1.97) | 1.79 (1.39 - 2.31) | 1.26 (0.83 - 1.89) |
| Other respiratory conditions | 1.21 (0.90 - 1.62) | 1.47 (1.31 - 1.64) | 1.22 (0.89 - 1.67) |
| Hyperbilirubinemia | 0.94 (0.76 - 1.16) | 1.22 (1.10 - 1.35) | 1.30 (1.03 - 1.64) |

^a^ Adjusted for maternal age, parity, and level of hospital care

^b^ The reference category was female infants

^c^ Respiratory distress syndrome of the newborn
